# A case of inguinal hernia associated with atypical *Dirofilaria repens* infection in a dog

**DOI:** 10.1186/s13071-021-04635-3

**Published:** 2021-02-26

**Authors:** Georgiana Deak, Angela Monica Ionică, Izabela Szasz, Marian Taulescu, Andrei Daniel Mihalca

**Affiliations:** 1 Department of Parasitology and Parasitic Diseases, University of Agricultural Sciences and Veterinary Medicine of Cluj-Napoca, Calea Mănăştur 3-5, Cluj-Napoca, Romania; 2 Molecular Biology and Veterinary Parasitology Unit (CDS 9), “Regele Mihai I al României” Life Science Institute, University of Agricultural Sciences and Veterinary Medicine of Cluj-Napoca, Calea Mănăştur 3-5, 400372 Cluj-Napoca, Romania; 3 SC Sabados Vet SRL, Arinului 4, 440186 Satu-Mare, Romania; 4 Department of Pathology, University of Agricultural Sciences and Veterinary Medicine of Cluj-Napoca, Calea Mănăştur 3-5, 400372 Cluj-Napoca, Romania

**Keywords:** *Dirofilaria repens*, Dog, Romania, Hernia

## Abstract

**Background:**

*Dirofilaria repens* is a filarioid nematode transmitted by mosquitoes. Adult *D. repens* are typically localized in the subcutaneous tissue of the host, but other, atypical localizations have also been reported. There have been several reports of clinical cases involving an association of parasites and hernias in both animals and humans. However, it is unclear if parasitic infection can act as a triggering factor in the development of hernias.

**Methods:**

A 12-year-old dog was referred to a private veterinarian clinic in Satu Mare, northwestern Romania due to the presence of a swelling in the lateral side of the penis (inguinal area). The dog underwent hernia repair surgery during which four long nematodes were detected in the peritoneal serosa of the inguinal hernial sac. One female specimen was subjected to genomic DNA extraction to confirm species identification, based on amplification and sequencing of a 670-bp fragment of the cytochrome* c* oxidase subunit 1 (*cox*1) gene. Treatment with a single dose of imidacloprid 10% + moxidectin 2.5% (Advocate, Bayer AG) was administered.

**Results:**

The nematodes were morphologically identified as adult *D. repens*, and the BLAST analyses revealed a 100% nucleotide similarity to a *D. repens* sequence isolated from a human case in Czech Republic.

**Conclusions:**

We report a case of an atypical localization of *D. repens* in the peritoneal cavity of a naturally infected pet dog with inguinal hernia and discuss the associations between hernia and parasitic infections.

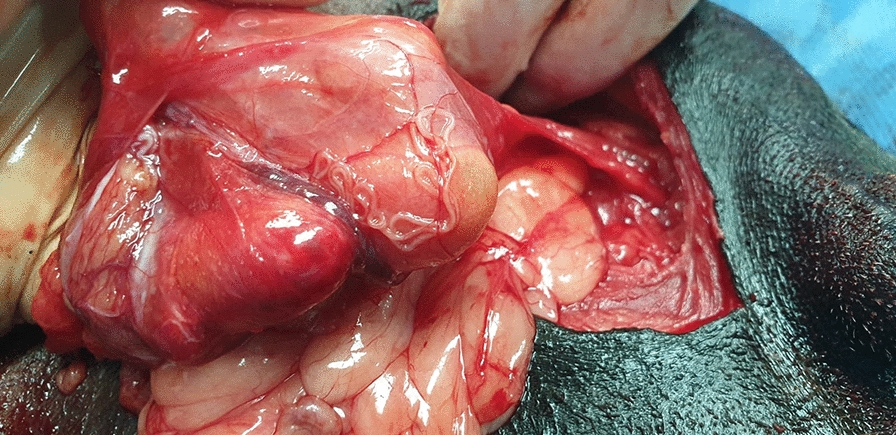

## Background

The genus* Dirofilaria* (Spirurida, Onchocercidae) includes vector-borne filarial nematodes with a worldwide distribution. Subcutaneous dirofilariasis is a common disease of both dogs and cats, but also of wild carnivores and humans [1]. * Dirofilaria repens* is transmitted by mosquitoes. Adult nematodes are typically localized in the subcutaneous tissues of the host where they freely move while microfilariae circulate in the blood stream where they are ingested by female mosquitoes [2–5]. Atypical localizations of the adult nematodes have also been reported [1, 6–9]. Infection with *D. repens* is associated with subcutaneous nodules, hyperpigmentation or, in most cases, no symptoms at all [10, 11]. Subclinically infected animals often remain undiagnosed due to the absence of any clinical signs, and they may represent important reservoirs for the spread of *D. repens* with zoonotic implications [12].

Several clinical cases involving an association of helminth parasites and hernias have been reported in both animals [6] and humans [13–16] (Table 1). However, it remains unclear whether parasitic infection can act as a triggering factor in the development of hernias. The authors of one study suggest that nematodes may act as an etiological agent of hernias [17], and several other studies have reported that certain nematodes can be responsible for hydrocele, which can in turn be complicated with inguinal hernia [18, 19].

**Table 1:** Review of studies on helminth-associated hernias worldwide

Parasite species	Host	Hernia location	Country	Reference
*Wuchereria bancrofti*	*Homo sapiens*	Inguinal	USA	[17]
*Wuchereria bancrofti*	*Homo sapiens*	Inguinal	Puerto Rico	[39]
*Wuchereria bancrofti*	*Homo sapiens*	Inguinal	UK	[40]
*Wuchereria bancrofti*	*Homo sapiens*	Inguinal	Ghana	[41]
*Wuchereria bancrofti*	*Homo sapiens*	Inguinal	France	[16]
*Dirofilaria immitis*	*Homo sapiens*	Inguinal	USA (California)	[14]
*Dirofilaria immitis*	*Homo sapiens*	Umbilical	UK	[13]
*Dirofilaria immitis*	*Canis familiaris*	Umbilical	Korea	[6]
*Dirofilaria immitis*	*Homo sapiens*	Inguinal	Iran	[42]
*Dirofilaria repens*	*Homo sapiens*	Inguinal	Italy	[38]
*Dirofilaria repens*	*Homo sapiens*	Inguinal	Czech Republic	[15]
*Dirofilaria repens*	*Canis familiaris*	Inguinal	Romania	Current paper
*Onchocerca* sp.	*Homo sapiens*	Inguinal, femoral	Kenya	[36]
*Anisakis* sp.	*Homo sapiens*	Inguinal	Japan	[43]
*Anisakis* sp.	*Homo sapiens*	Epigastric	Canada	[44]
*Pseudoterranova azarasi*	*Homo sapiens*	Inguinal	Japan	[23]
*Schistosoma japonicum*	*Homo sapiens*	Inguinal	China	[45]
*Schistosoma japonicum*	*Homo sapiens*	Inguinal	Taiwan	[34]
*Schistosoma mansoni*	*Homo sapiens*	iIguinal	USA (Philadelphia)	[46]
*Paragonimus westermanii*	*Homo sapiens*	Inguinal	Japan	[47]
*Paragonimus westermanii*	*Homo sapiens*	Inguinal	Korea	[48]
*Echinococcus granulosus*	*Homo sapiens*	Inguinal	India	[49]
*Echinococcus granulosus*	*Homo sapiens*	Inguinal	Iran	[50]
*Armillifer armillatus*	*Homo sapiens*	Inguinal	Benin	[51]

The aim of this study was to describe a case of an atypical subclinical infection with *D. repens* in the peritoneal cavity of a naturally infected pet dog with inguinal hernia and to review and discuss the associations between hernia and parasitic helminth infections.

## Methods

**Fig. 1:**
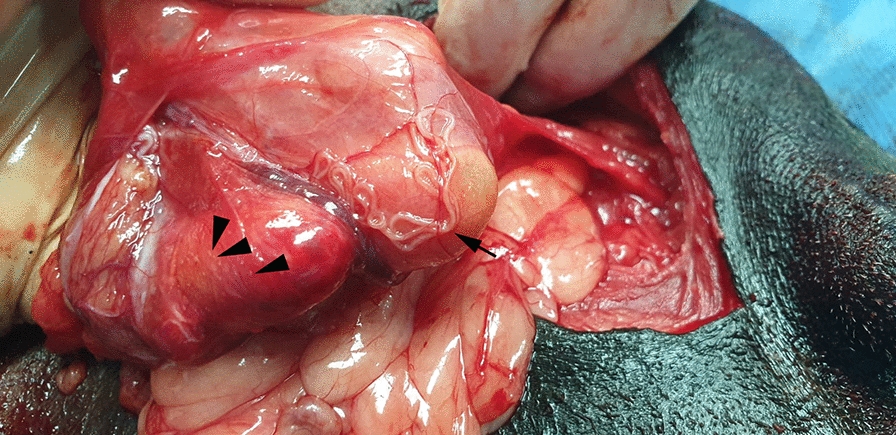
Arrow indicates adult *Dirofilaria repens* worms in the hernia sac. Arrowhead indicates white nodules disseminated on the surface of the peritoneum.

A 12-year-old male, mixed breed dog was referred to a private clinic (Sabados Vet) in the city of Satu Mare, north-western Romania, on 24 April 2020 due to the presence of a swelling in the postero-lateral side of the penis (inguinal area). The dog was housed indoors, with daily access to the outside environment. A complete clinical examination was performed, and no other health issues were detected, other than what proved to be an inguinal hernia when palpated. An abdominal ultrasound examination was carried out to elucidate the cause of the swelling and a blood sample was collected for a complete laboratory evaluation. The animal underwent hernia repair surgery the following day during which four long nematodes were detected on the surface of the peritoneal serosa of the inguinal hernial sac. Grossly, the affected tissue was diffusely and moderately congested and thickened and showed numerous, prominent and white nodules disseminated on the surface of the peritoneum, consisting of lymphoid cells aggregates (milky spots). The non-strangulated hernial sac contained portions of small intestine and greater omentum. No significant free fluid was observed at this level (Fig. 1).

 The nematodes were collected and stored in absolute ethanol and sent together with a blood sample collected in EDTA tubes to the Department of Parasitology and Parasitic Diseases (Faculty of Veterinary Medicine of Cluj-Napoca) for morphological and molecular identification of the adult nematodes and for a modified Knott’s test, respectively. The collected nematodes were morphologically identified using descriptions provided in [20]. One female specimen was subjected to genomic DNA extraction in order to confirm species identification, based on amplification and sequencing of a 670-bp fragment of the cytochrome* c* oxidase subunit 1 gene (*cox*1), as previously described [21]. Genomic DNA was also isolated from 200 μl of whole blood and further processed by means of multiplex PCR [22] to exclude other species of blood-circulating microfilariae which are known to be present in Romania.

After the identification of the nematodes, the dog was treated with a single dose of imidacloprid 10% + moxidectin 2.5% (Advocate; Bayer AG, Leverkusen, Germany) in the clinic and the owner was advised to repeat the treatment monthly. Unfortunately, the dog had not been a regular patient of the clinic, with the present case being the first time it had been examined at the clinic; no information on past routine deworming and ectocide treatments was available.

Six months after the initial worm treatment, the dog was referred to the clinic for a control examination, and a second blood sample was collected to evaluate the efficacy of the treatment using Knott’s test. The owner was advised to use insect repellents at monthly intervals as prophylactic treatment.

## Results

The abdominal ultrasound performed at the first visit did not reveal any specific abnormalities, with the exception of an inguinal hernia. Blood analyses showed a total white blood cell count of 15.15 thousands/mm^3^ (reference interval [RI] 6–17), lymphocytosis (38.0%; RI 10.0–30.0%), monocytosis (14.9%; RI 2–10%), eosinophilia (11.2%; RI 1.6–7.5%) and neutropenia (35.6%; RI 50.0–80.0%). The results of all biochemical tests were within normal values, except for moderate hypokalemia (3.1 mmol/l; RI 3.5–5.6 mmol/L).

**Fig. 2:**
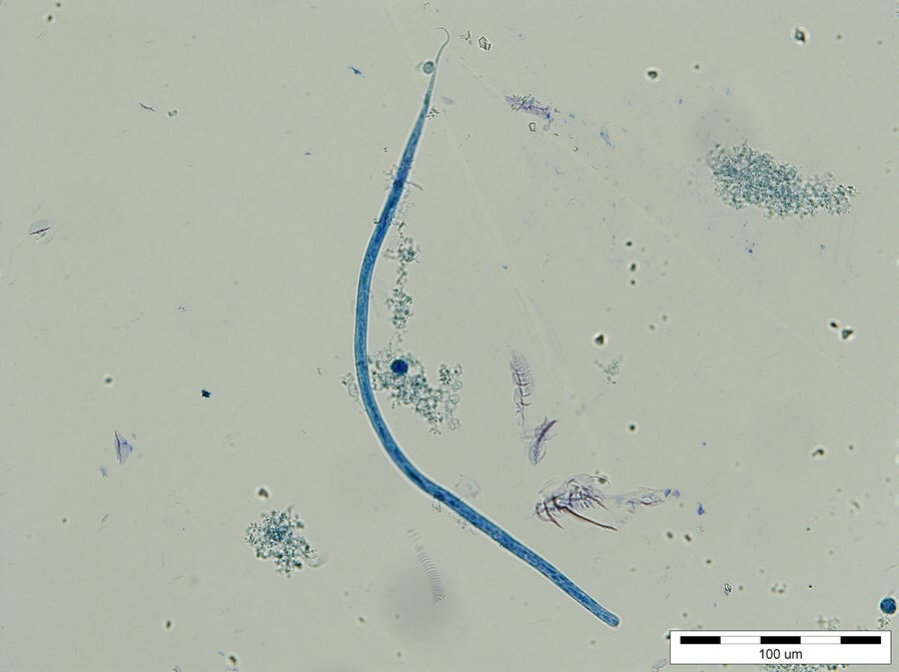
*Dirofilaria repens* microfilariae using the Knott’s test

All nematodes were morphologically identified as *D. repens* adults (two males and two females). The specimens presented a striated cuticle with longitudinal ridges on the surface. The female nematodes were 4.9–6.1 mm wide and 15.3–17.1 cm long; the males were 3.9–4.2 mm wide and 6.4–7.2 cm long. The Knott’s test revealed the presence of microfilariae morphologically identified as *D. repens* (Fig. 2). The microfilariae were 5.8–7.8 µm wide and 330–374 µm long and had a rounded anterior extremity and well-developed sub-cephalic space; the caudal extremity was generally curved. No co-infection with *D. immitis* or *Acanthocheilonema reconditum* was observed microscopically, nor detected by multiplex PCR. The BLAST analyses revealed a 100% nucleotide similarity to a *D. repens* sequence isolated from a human case in Czech Republic (Accession number KR998257). Our sequence was deposited in GenBank under the accession number MW065790. After 6 months, the dog had no visible cutaneous nodules, but the second blood sample collected at the same time was still positive for *D. repens* because the owner had neglected to follow the clinic’s recommendation to repeat the treatment monthly.

## Discussion

Inguinal hernias in mammals are very common and may be produced by traumatic factors, intense effort, or in association with a congenital background. The presence of parasitic infections associated with hernias has been reported previously. Among the parasites associated with hernias, filarial parasites are the most common. In Africa and the Americas, the nematode most frequently associated with hernias is *Wuchereria bancrofti*, while in Europe, most of the parasite–hernias associations reported have involved *Dirofilaria* spp. (Table 1). There has also been a recent report of an extra-gastrointestinal anisakidosis caused by *Pseudoterranova azarasi* that manifested as strangulated inguinal hernia: the presence of nematodes within peritoneal serosa of the inguinal hernia sac was associated with severe granulomatous inflammation and numerous eosinophils [23]. In our case, during surgery we macroscopically detected a localized serosal inflammatory reaction with severe edema, possibly suggesting a role of filarial-induced peritoneal inflammation in the development of inguinal hernia. The edematous changes with disruption of the collagen fibers in the submesothelium can be caused by the direct effect of the parasites and/or by an impairment and dysfunction of lymphatic drainage. Evidence of numerous and prominent “milky spots” on the surface of the affected peritoneal serosa is another sign of peritoneal inflammation [24]. Enlargement of the inguinal and subinguinal lymph nodes, lymphangitis, lymphangiectasia and scrotal edema are common signs of the presence of filarial parasites in human patients [17]. However, we did not observe any changes to the lymphatic system in our canine patient. Visceral infections with protozoan organisms, including *Leishmania donovani* [25] and *Toxoplasma gondii* [26], have been reported to be associated with hernias in humans. In these cases, the abdominal hernia was likely caused by (i) protozoan infection-related hepatomegaly and splenomegaly, resulting in increased abdominal pressure; (ii) alterations to the skeletal muscles (e.g. degeneration and disruption) of the abdominal wall; or (iii) changes to the peritoneal serosa. *Dirofilaria immitis* is a well-known parasite and is responsible for severe symptoms in dogs, while *D. repens* is occasionally produces mild dermatological lesions [27]. Humans can be accidental hosts when they are infected by *D. repens*, but these nematodes do not usually reach maturity in humans and they erratically migrate through the body to form subcutaneous nodules [28–31]. However, on occasion *D. repens* can produce atypical and severe lesions, as described in a case from a human patient in Romania [32].

Even though it is still only a hypothesis that parasites may play a role in the pathophysiology of hernias, our case involves the detections of a *D. repens* infection during surgery for a non-strangulated inguinal hernia. Knott [17] suggested that filarial hydrocele can produce an inguinal hernia due to the weight of the sac, impaired lymph circulation and lengthening of the cord, which can dilate the inguinal canal and drag down the peritoneal serosa [17]. Another theory is that a parasitic infection could induce granulomatous or muscular pathological reactions in the host [33] and that these may be responsible for collagen degeneration, resulting in an inguinal hernia [34]. Rodhain [35] considered that hanging groins are produced by allergic reactions to microfilariae of *O. volvulus*, a condition that is responsible for producing complications such as a hernia [35, 36].

In the case of our canine patient, we recommended prophylactic treatment, even though the locality is not considered to be a risk area for infection with heartworms, although this may be due to the absence of studies in this field in northwestern Romania [26, 36]. Both veterinarians and medical doctors should consider dirofilariasis as a diagnostic option, especially in endemic areas. It should be noted that the clinical aspect of this disease is not always typical and may lead to severe complications, even in dog owners [8, 32, 37, 38].

## Conclusions

This is the first report of a parasite-associated inguinal hernia in a dog. The relatively numerous publications on helminth-associated hernias, as well as other pathophysiological effects, suggest a possible involvement of parasites in this surgical condition in humans and dogs. The present case also extends the known geographical distribution of *D. repens* in Romania and highlights the importance of promoting control and prophylactic measures by reducing the mosquito’s populations and treating infected dogs which can serve as reservoir hosts. These measures are highly important in order to minimize the transmission to other hosts, given the zoonotic potential of *D. repens*.

## Data Availability

Not applicable.
